# Hospital physicians´ working hour characteristics and sleep quality: a cross-sectional analysis of realized working hour and survey data

**DOI:** 10.1186/s12913-022-08336-0

**Published:** 2022-07-23

**Authors:** Kati Karhula, Aki Koskinen, Jenni Ervasti, Tarja Hakola, Veli-Matti Isoviita, Ilkka Kivimäki, Sampsa Puttonen, Tuula Oksanen, Mikko Härmä

**Affiliations:** 1 Finnish Institute of Occupational Health, P.0. Box 40, FI-00032 Työterveyslaitos, Helsinki, Finland; 2 Faculty of Medicine, University of Helsinki, Helsinki, Finland; 3 Faculty of Social Sciences, Tampere University, Tampere, Finland; 4 Institute of Public Health and Clinical Nutrition, University of Eastern Finland, Kuopio, Finland

**Keywords:** On-call work, Payroll data, Working time, Sleep quality, Sleep difficulties

## Abstract

**Background:**

Hospital physicians’ work includes on-call duties to provide 24/7 health care. Previous studies using self-reported survey data have associated long working hours and on-call work with sleep difficulties. To reduce recall bias, we complemented survey data with payroll-based objective data to study whether hospital physicians’ realized working hours are associated with sleep.

**Methods:**

The study was nested within the Finnish Public Sector study. We used survey data on 728 hospital physicians (mean age 43.4 years, 62% females) from 2015 linked to realized daily working hour data from 3 months preceding the survey. The associations of working hour characteristics with sleep quantity and quality were studied with multinomial logistic regression analysis adjusted for demographics, overall stressfulness of life situation, control over scheduling of shifts, and hospital district.

**Results:**

One fourth (26%) of the participants reported short (≤6.5 h) average sleep duration. Frequent night work (> 6 shifts/91 days) was associated with short sleep (OR 1.87 95%CI 1.23–2.83) compared to no night work. Approximately one third (32%) of the physicians reported insufficient sleep. Physicians with long weekly working hours (> 48 hours) had higher odds for insufficient sleep (OR 1.78 95%CI 1.15–2.76) than physicians with short weekly working hours (< 40 hours). Insufficient sleep was also associated with frequent on-call duties (> 12 shifts/3 months OR 2.00 95%CI 1.08–3.72), frequent night work (OR 1.60 95%CI 1.09–2.37), and frequent short shift intervals (≤11 hours; > 12 times/3 months OR 1.65 95%CI 1.01–2.69) compared to not having these working hour characteristics. Nearly half of the physicians (48%) reported at least one sleep difficulty at least two times a week and frequent night work increased odds for difficulties in initiating sleep (OR 2.43 95%CI 1.04–5.69). Otherwise sleep difficulties were not associated with the studied working hour characteristics.

**Conclusion:**

We used realized working hour data to strengthen the evidence on on-call work and sleep quality and our results advice to limit the frequency of night work, on-call shifts, short shift intervals and long weekly working hours to promote hospital physicians’ sufficient sleep.

**Supplementary Information:**

The online version contains supplementary material available at 10.1186/s12913-022-08336-0.

## Introduction

In surveys, hospital physicians report having worked long weekly hours, often over 50 hours per week [1–4] and in some cases, clearly above 60 hours per week [5]. As hospitals operate 24 hours 7 days a week to provide specialized health care services, hospital physicians need to work also during evenings and weekends. Approximately 70% of hospital physicians have on-call duties in addition to their regular working hours, or they provide consultation while not on duty (on-call at home) [2, 6, 7]. In previous studies, majority of all physicians have reported at least four on-call shifts per month [6, 8, 9]. In a large German survey of nearly 2000 hospital physicians, approximately 15% of them reported having had at least nine on-call shifts at the workplace per month [6].

On-call work often involves both long working hours and night-time duties, intense work pace [10, 11], rapid decision-making [10] and treating patients with severe or life-threatening conditions [10, 12]. Physicians’ extensive working hours are associated with, for example, burnout [13, 14] and therefore arrangements of on-call work are important to the physicians´ satisfaction and wellbeing. Only a half of surgeons were satisfied with their on-call duty arrangements and dissatisfaction increased by the age of the surgeon [15]. On-call work has been associated with self-reported adverse effects such as sleeping problems [8, 16, 17] and work-family interference [17, 18]. Among anaesthesiologists, approximately 20% experienced non-restorative sleep, and frequency of on-call work increased the likelihood of insomnia [8]. As in other professions with long and/or irregular working hours, habitual insufficient sleep duration is a long-term health concern also among physicians [12]. Although the health effects of on-call work have not yet been widely studied, previous research shows an association of on-call work with, for example, functional gastrointestinal disorders [19].

On-call work also influences turnover intentions: the highest levels of turnover intentions were among those physicians who had both on-call duties and experienced a high level of job strain [20]. On-call duties may also have negative effects to patient safety. A higher complication rate in surgical operations after night duties has been observed when the surgeon has less than 6 h of rest between work shifts compared to 6 or more hours of rest [21].

On-call work is a less studied working hour dimension than shift work and long working hours [22], and it seems that on-call work at home has less research interest. In a recent review [23], on-call work at home was associated with decreased sleep duration, and in most cases, poor sleep quality. Among German physicians and other health care employees, on-call work at home was associated with increased risk of sleep difficulties despite low inclusion criteria (≥1 shift/month) for having on-call at home [24]. Physicians typically receive 2–3 phone calls during the night [10] when in on-call work at home.

Physicians’ working hours have been collected with diaries for shorter time periods [25], but studies based on payroll data are lacking. Moreover, in previous studies, it has seldom been clearly described whether on-call work refers only to on-call work at the workplace, on-call at home or both. There are thus no previous studies that have utilized payroll-based data on both regular working hours and on-call work when studying physicians sleep. Self-reported working hour data is prone to recall bias [26] and to narrow this research gap, we utilized register-based working hour data. The aim of this study is to investigate the association of hospital physicians’ working hour characteristics with sleep, i.e., self-reported sleep length, insufficient sleep, and occurrence of sleep difficulties.

## Methods

### Study sample and participants

The study participants were hospital physicians from four hospital districts participating in the Finnish Public Sector (FPS) study survey in 2015 (*N* = 11,274, response rate 69%, physicians *n* = 1018). Physicians were identified from other occupational groups by working time regime coding in registry-based working hour data. Similarly to our previous study [27], physicians’ regular working hour data was retrieved from the working time scheduling program Titania® and the on-call working hour data from Titania for physicians® software (CGI Finland Ltd., Helsinki, Finland). Altogether 728 physicians had combined data from survey and the two working time scheduling software.

### Register-based working hour data

The working hour data included realized payroll-based starting and ending times for each work period and absences including days off, sickness absences and other leaves. The working hour characteristics were created from 3 months (91 days) preceding the survey and the data included both regular working hours and all on-call hours at the workplace. The register-data included also starting and ending times for each period of on-call work at home, but this part of data was utilized separately, as these duties are carried out more commonly by senior physicians [6]. The cut-points in the studied working hour characteristics were based on the European Working Time directive [28], the Finnish Institute of Occupational Health (FIOH) recommendations on ergonomic working times [29] and, if no regulatory or research-based cut-points were available, to the distribution of the data utilized.

### Survey-based working hours

Average total weekly working hours were asked with a question “On average, how much time do you spend performing your work tasks (normal working time, overtime at workplace and at home and including subsidiary employment)?” and answered as hours per week.

### Sleep measures

Average 24-hour *sleep duration* was surveyed with the question “How many hours do you normally sleep during 24 hours?” with options from 5 h or less to 10 h or more in 30-minute intervals [30]. Short sleep was dichotomized as ≤6.5 hours [31]. The *insufficient sleep* item was derived from the question “Do you sleep enough?” with alternatives “yes, always or nearly always”, “yes, often”, and “seldom or never”. *Sleep difficulties* (difficulties to fall asleep, waking up several times per night, difficulties in staying asleep, and feeling tired and worn out after waking up after usual amount of sleep; from now on ‘non-restorative sleep‘, for brevity) during the last 4 weeks were enquired with the Jenkins Sleep Scale ranging from “not at all” to “every day” [32]. The six categorized answer options (not at all - 1-3 nights/month, approx. 1 night/wk., 2–4 nights/wk., 5–6 nights/wk., almost every night) were dichotomized as having the sleep difficulties if the frequency was 2–4 times per week or more often.

### Covariates

In addition to age, sex, number of children and hospital district, the analyses were adjusted for control over scheduling of shifts and overall stressfulness of life situation. The first was assessed with the item “How much control do you have over scheduling of work shifts?” [33] with five options from “very much” to “very little”. The latter was used as a 6-point Likert-type scale, from “easy” to “extremely burdensome”.

### Ethical issues

All the hospital districts gave written permission to FIOH to use the employers´ working time registers for research. All data was pseudonymized, and international ethical standards were conformed. The coordinating ethics committee of the Hospital District of Helsinki and Uusimaa (HUS) have approved the FPS study (HUS 1210/2016).

### Statistical methods

The statistical analyses were conducted using IBM SPSS Statistics 27 (IBM Corp., Armonk, NY, USA). The associations of the studied working hour characteristics and sleep variables (duration, sufficiency, sleep difficulties) were studied with multinomial regression analysis. After crude analysis, we adjusted the models first with age, gender and hospital district as covariates, followed by models with age, sex, marital status, having children under 18 years, overall stressfulness of life situation, and hospital district as covariates and finally, the fully adjusted models with age, sex, marital status, having children under 18 years, overall stressfulness of life situation, control over scheduling of shifts, and hospital district. As there were missing values in the responses of having children under 18 years, the missing values comprised their own class.

## Results

### Participants’ characteristics

The physicians’ mean age was 43.4 years (SD 9.9, range 26–68 years), and 62% (*n* = 453) of them were female. The physicians had worked in their current workplace (i.e., hospital district) on average for 9.5 years (SD 8.3, range 0–38 years) and in their current position on average for 5.1 years (SD 5.4, range 0–33 years).

### Working hour characteristics

In the 3 months preceding the survey, the average realized weekly working hours were approximately 38 hours per week when realized regular working time and on-call work at workplace were combined. However, in the FPS survey, the physicians had estimated their total weekly working hours at 45.4 h (SD 9.3 h) when also on-call at home and possible second jobs were included. According to the realized working hour data, the physicians had five night duties on average, 2.5 weekend shifts and seven short shift intervals (≤11 h) during the 3 months. The mean length of on-call shifts was 14.5 hours, however only 44 physicians had at least one 24-hour or longer on call-shift. Approximately 45% (*n* = 369) of the physicians had on-call work at home with a mean of 24.5 hours per week. (Table 1).

**Table 1:** Hospital physicians´ average working hour characteristics in the 3 months preceding the FPS 2015 survey

	N	Mean	SD	Min	Max
Working hours/week^a^	728	37.81	10.98	3.00	73.95
Number of on-call shifts^a,b^	403	7.77	6.87	1	63
Length of on-call shifts (hours) ^b^	403	14.49	5.12	1.70	25.00
Number of < 12-hour on-call shifts^b^	403	2.99	6.35	0	63
Number of 12–24-hour on-call shifts^b^	403	4.62	3.66	0	20
Number of > 24-hour on-call shifts^b^	403	0.17	0.56	0	4
Number of night work duties ^a,b,c^	403	5.29	4.01	0	20
Number of weekend work duties^a,b,d^	403	2.54	1.84	0	10
Number of ≤11-hour shift intervals^a,b^	403	7.08	7.56	0	52
On-call work at home hours/week^a,e^	372	24.55	12.55	0.42	72.67

^a^working hour characteristic included in the analysis

^b^among physicians having at least one on-call shift at workplace

^c^at least 3 hours of work between 23 and 06

^d^at least 3 hours of work between Fri 18:00 and Mon 08:00

^e^among physicians having at least one on-call shift at home

### Sleep duration

One fourth (26%, *n* = 187) of the participants had short (≤6.5 hours) self-reported sleep duration, male physicians more often than female physicians (30% vs. 23%, *p* < 0.03). High numbers of night work shifts (≥7 shifts/3 months) and estimated long (> 48 h) weekly total working hours were associated with higher odds for insufficient sleep (OR 1.87, 95%CI 1.23–2.83; OR 2.09, 95%CI 1.25–3.94, respectively), whereas having less than an average amount of on-call work at home was associated with lower odds for insufficient sleep compared to no on-call work at home (OR 0.64, 95%CI 0.41–0.99). (Table 2).

**Table 2:** The associations of physicians´ working hour characteristics from 3 months with short sleep duration (≤6.5 h)

		Crude model^a^			Adjusted model^a,b^		
		N	OR	95%CI	N	OR	95%CI
Average working hours /week	< 40	450	1		436	1	
	40–48	150	1.20	(0.79–1.82)	144	1.39	(0.90–2.16)
	> 48	120	1.23	(0.81–1.99)	119	1.59	(0.99–2.56)
Number of on-call shifts	0	427	1		414	1	
	1–12	346	0.90	(0.65–1.24)	337	1.15	(0.81–1.65)
	≥13	55	0.93	(0.49–1.76)	55	1.35	(0.68–2.69)
Number of night work duties^c^	0	503	1		489	1	
	1–3	56	0.75	(0.38–1.49)	54	0.83	(0.40–1.73)
	4–6	82	1.12	(0.66–1.90)	80	1.55	(0.89–2.71)
	≥7	187	1.37	(0.95–1.99)	183	1.87	(1.23–2.83)
Number of weekend work duties^d^	0	483	1		468	1	
	1–4	234	0.77	(0.53–1.12)	227	1.03	(0.69–1.53)
	≥5	52	1.35	(0.73–2.49)	52	1.93	(0.99–3.74)
Number of ≤11-hour shift intervals	0	520	1		505	1	
	1–6	111	0.69	(0.42–1.13)	108	0.81	(0.49–1.35)
	7–12	97	0.92	(0.56–1.50)	94	1.20	(0.71–2.04)
	> 12	92	0.88	(0.53–1.46)	91	1.09	(0.63–1.89)
On-call work at home hours/week	0	459	1		449	1	
	1–20	181	0.77	(0.51–1.16)	175	0.64	(0.41–0.99)
	> 20	188	1.16	(0.79–1.68)	182	1.01	(0.68–1.49)

^a^multinomial logistic regression analysis

^b^adjusted for age, gender, marital status, number of children, overall stressfulness of the life situation, control over working times and hospital district

^c^at least 3 hours of work between 23 and 06

^d^at least 3 hours of work between Fri 18:00 and Mon 08:00

### Insufficient sleep

Approximately one third (32%) of the physicians reported insufficient sleep, without gender difference (*p* = 0.51). Of those who reported having insufficient sleep, 62% had a short self-reported sleep duration (≤6.5 hours, *p* < 0.001). Realized and estimated long (> 48 h) weekly working hours were both associated with insufficient sleep (OR 1.78 95%CI 1.15–2.76; OR 2.12 95%CI 1.30–3.45, respectively). The other working hour characteristics with higher odds for insufficient sleep were high (≥13/3 months) number of on-call shifts (OR 2.00 95%CI 1.08–3.72), frequent (≥7/3 months) night work (OR 1.60 95%CI 1.09–2.37), and high number (> 12/3 months) of max 11-hour shift-intervals (OR 1.65 95%CI 1.01–2.69). (Table 3).

**Table 3:** The associations of physicians´ working hour characteristics from 3 months with insufficient sleep

		Crude model^a^			Adjusted model^a,b^		
		N	OR	95%CI	N	OR	95%CI
Average working hours/week	< 40	450	1		435	1	
	40–48	148	1.35	(0.90–2.01)	142	1.32	(0.88–2.00)
	> 48	120	1.79	(1.18–2.73)	118	1.78	(1.15–2.76)
Number of on-call shifts	0	417	1		405	1	
	1–12	339	1.28	(0.93–1.75)	330	1.17	(0.83–1.65)
	≥13	55	1.93	(1.09–3.45)	55	2.00	(1.08–3.72)
Number of night work duties^c^	0	490	1		477	1	
	1–3	56	0.73	(0.38–1.43)	54	0.72	(0.37–1.43)
	4–6	81	1.58	(0.96–2.59)	79	1.55	(0.93–2.59)
	≥7	184	1.65	(1.15–2.36)	180	1.60	(1.09–2.37)
Number of weekend work duties^d^	0	472	1		458	1	
	1–4	229	1.27	(0.90–1.78)	222	1.22	(0.84–1.75)
	≥5	51	1.55	(0.85–2.82)	51	1.39	(0.74–2.60)
Number of ≤11-hour shift intervals	0	509	1		495	1	
	1–6	108	1.17	(0.74–1.84)	105	1.05	(0.66–1.68)
	7–12	94	1.65	(1.04–2.26)	91	1.56	(0.96–2.53)
	> 12	92	1.71	(1.08–2.72)	91	1.65	(1.01–2.69)
On-call work at home hours/week	0	452	1		443	1	
	1–20	174	0.72	(0.49–1.08)	168	0.73	(0.48–1.09)
	> 20	185	0.95	(0.66–1.38)	179	1.95	(0.65–1.39)

^a^multinomial logistic regression analysis

^b^adjusted for age, gender, marital status, number of children, overall stressfulness of the life situation, control over working times and hospital district

^c^at least 3 hours of work between 23 and 06

^d^at least 3 hours of work between Fri 18:00 and Mon 08:00

### Sleep difficulties

Nearly half of the physicians (48%, *n* = 347) experienced often at least one sleep difficulty, female physicians more often than males (52% vs. 43%, *p* < 0.02). Of those who reported insufficient sleep, 84% reported having also at least one sleep difficulty (*p* < 0.001). However, none of the studied working hour characteristics were associated with having at least one sleep difficulty (Table 4). However, when the odds for each sleep difficulty with regards to the working hour characteristics were studied separately, frequent night work increased odds for difficulties in initiating sleep (OR 2.43 95%CI 1.04–5.69) (Table 5).

**Table 4:** The associations of physicians´ working hour characteristics from 3 months with having at least one sleep difficulty

		Crude model^a^			Adjusted model^a,b^		
		N	OR	95%CI	N	OR	95%CI
Average working hours/week	< 40	450	1		436	1	
	40–48	149	1.09	(0.75–1.58)	143	1.09	(0.74–1.60)
	> 48	121	0.72	(0.48–1.08)	120	0.69	(0.45–1.05)
Number of on-call shifts	0	425	1		412	1	
	1–12	346	0.97	(0.73–1.29)	337	1.03	(0.76–1.41)
	≥13	55	1.03	(0.58–1.81)	55	0.87	(0.48–1.60)
Number of night work duties^c^	0	502	1		488	1	
	1–3	56	0.89	(0.51–1.54)	54	0.85	(0.47–1.50)
	4–6	82	0.79	(0.49–1.25)	80	0.80	(0.49–1.32)
	≥7	186	0.94	(0.67–1.32)	182	0.95	(0.65–1.37)
Number of weekend work duties^d^	0	481	1		466	1	
	1–4	234	0.99	(0.72–1.36)	227	1.03	(0.73–1.44)
	≥5	52	1.30	(0.73–2.32)	52	1.34	(0.72–2.48)
Number of ≤11-hour shift intervals	0	518	1		503	1	
	1–6	111	1.05	(0.70–1.59)	108	1.12	(0.73–1.72)
	7–12	98	0.77	(0.50–1.18)	95	0.82	(0.52–1.30)
	> 12	92	0.87	(0.56–1.35)	91	0.85	(0.53–1.37)
On-call work at home hours/week	0	458	1		448	1	
	1–20	181	1.05	(0.74–1.48)	176	1.08	(0.75–1.55)
	> 20	187	0.84	(0.62–1.23)	180	0.86	(0.60–1.23)

^a^multinomial logistic regression analysis

^b^adjusted for age, gender, marital status, number of children, overall stressfulness of the life situation, control over working times and hospital district

^c^at least 3 hours of work between 23 and 06

^d^at least 3 hours of work between Fri 18:00 and Mon 08:00

**Table 5:** The associations of physicians´ working time characteristics from 3 months with often having difficulties to fall asleep

		Crude model^a^			Adjusted model^a,b^		
		N	OR	95%CI	N	OR	95%CI
Average working hours/week	< 40	450	1		437	1	
	40–48	151	1.16	(0.59–2.27)	145	1.07	(0.54–2.12)
	> 48	120	2.30	(0.89–5.97)	119	2.12	(0.81–5.59)
Number of on-call shifts	0	427	1		415	1	
	1–12	347	1.30	(0.77–2.19)	338	1.36	(0.78–2.40)
	≥13	55	5.43	(0.73–40.32)	55	5.14	(0.67–39.13)
Number of night work duties^b^	0	504	1		491	1	
	1–3	56	1.73	(0.52–5.77)	54	1.68	(0.50–5.68)
	4–6	83	0.81	(0.38–1.72)	81	0.79	(0.36–1.73)
	≥7	186	2.18	(1.01–4.72)	182	2.43	(1.04–5.69)
Number of weekend work duties^c^	0	483	1		469	1	
	1–4	236	1.48	(0.80–2.71)	229	1.39	(0.73–2.62)
	≥5	52	1.64	(0.49–5.47)	52	1.51	(0.44–5.19)
Number of ≤11-hour shift intervals	0	520	1		506	1	
	1–6	112	0.87	(0.44–1.74)	109	0.85	(0.42–1.73)
	7–12	98	1.76	(0.68–4.56)	95	1.75	(0.66–4.64)
	> 12	91	2.06	(0.72–5.88)	90	1.94	(0.66–5.69)
On-call work at home hours/week	0	374	1		369	1	
	1–20	240	1.31	(0.69–2.49)	235	1.30	(0.67–2.49)
	> 20	228	0.82	(0.46–1.45)	227	0.79	(0.44–1.42)

^a^multinomial logistic regression analysis

^b^adjusted for age, gender, marital status, number of children, overall stressfulness of the life situation, control over working times and hospital district

^c^at least 3 hours of work between 23 and 06

^d^at least 3 hours of work between Fri 18:00 and Mon 08:00

In the crude models having frequent (≥5/3 months) weekend work increased odds for waking up several times per night (OR 2.40 95%CI 1.06–5.45) (Table 6) and having frequent (≥7/3 months) night work increased odds for having difficulties in staying asleep (OR 1.64 95%CI 1.00–2.68) (Table 7). There were no associations between the studied working hour characteristics and non-restorative sleep (Table 8).

**Table 6:** The associations of physicians´ working time characteristics from 3 months with often waking up several times per night

		Crude model^a^			Adjusted model^a,b^		
		N	OR	95%CI	N	OR	95%CI
Average working hours/week	< 40	450	1		436	1	
	40–48	149	1.05	(0.69–1.61)	143	1.04	(0.66–1.62)
	> 48	120	0.78	(0.50–1.21)	119	0.70	(0.43–1.11)
Number of on-call shifts	0	426	1		413	1	
	1–12	346	1.13	(0.82–1.56)	337	1.03	(0.72–1.46)
	≥13	55	1.52	(0.76–3.02)	55	1.08	(0.52–2.24)
Number of night work duties^b^	0	503	1		489	1	
	1–3	56	0.88	(0.48–1.63)	54	0.76	(0.40–1.43)
	4–6	82	0.96	(0.57–1.63)	80	0.89	(0.51–1.55)
	≥7	186	1.07	(0.73–1.58)	182	0.94	(0.61–1.45)
Number of weekend work duties^c^	0	482	1		467	1	
	1–4	235	1.02	(0.72–1.45)	228	0.91	(0.62–1.34)
	≥5	52	2.40	(1.06–5.45)	52	2.26	(0.96–5.34)
Number of ≤11-hour shift intervals	0	519	1		504	1	
	1–6	112	1.30	(0.80–2.13)	109	1.28	(0.77–2.13)
	7–12	97	0.83	(0.52–1.34)	94	0.80	(0.48–1.34)
	> 12	92	1.01	(0.61–1.67)	91	0.91	(0.52–1.57)
On-call work at home hours/week	0	460	1		450	1	
	1–20	180	0.68	(0.46–1.00)	174	0.73	(0.49–1.09)
	> 20	187	0.82	(0.56–1.21)	181	0.86	(0.58–1.28)

^a^multinomial logistic regression analysis

^b^adjusted for age, gender, marital status, number of children, overall stressfulness of the life situation, control over working times and hospital district

^c^at least 3 hours of work between 23 and 06

^d^at least 3 hours of work between Fri 18:00 and Mon 08:00

**Table 7:** The associations of physicians´ working time characteristics from 3 months with often having difficulties in staying asleep

		Crude model^a^			Adjusted model^a,b^		
		N	OR	95%CI	N	OR	95%CI
Average working hours/week	< 40	448	1		434	1	
	40–48	150	1.21	(0.74–1.99)	144	1.16	(0.69–1.95)
	> 48	121	1.32	(0.76–2.30)	120	1.14	(0.63–2.07)
Number of on-call shifts	0	424	1		411	1	
	1–12	346	1.46	(1.00–2.15)	337	1.15	(0.76–1.74)
	≥13	55	1.45	(0.66–3.19)	55	0.80	(0.35–1.86)
Number of night work duties^c^	0	500	1		486	1	
	1–3	56	1.39	(0.64–3.04)	54	1.15	(0.51–2.57)
	4–6	83	0.90	(0.50–1.60)	81	0.73	(0.40–1.34)
	≥7	186	1.64	(1.00–2.68)	182	1.21	(0.71–2.06)
Number of weekend work duties^d^	0	480	1		465	1	
	1–4	234	1.16	(0.76–1.76)	227	0.88	(0.56–1.38)
	≥5	52	1.72	(0.71–4.16)	52	1.24	(0.49–3.12)
Number of ≤11-hour shift intervals	0	517	1		502	1	
	1–6	110	0.97	(0.57–1.66)	107	0.91	(0.53–1.59)
	7–12	98	1.03	(0.58–1.82)	95	0.82	(0.45–1.51)
	> 12	92	1.32	(0.70–2.47)	91	1.05	(0.53–2.08)
On-call work at home hours/week	0	457	1		447	1	
	1–20	182	0.88	(0.56–1.39)	176	0.97	(0.60–1.55)
	> 20	186	0.73	(0.47–1.13)	180	0.84	(0.53–1.32)

^a^multinomial logistic regression analysis

^b^adjusted for age, gender, marital status, number of children, overall stressfulness of the life situation, control over working times and hospital district

^c^at least 3 hours of work between 23 and 06

^d^at least 3 hours of work between Fri 18:00 and Mon 08:00

**Table 8:** The associations of physicians´ working time characteristics from 3 months with often having non-restorative sleep

		Crude model^a^			Adjusted model^a,b^		
		N	OR	95%CI	N	OR	95%CI
Average working hours/week	< 40	451	1		437	1	
	40–48	151	1.09	(0.74–1.61)	145	1.13	(0.75–1.71)
	> 48	120	0.79	(0.52–1.19)	119	0.79	(0.51–1.22)
Number of on-call shifts	0	428	1		415	1	
	1–12	348	1.10	(0.82–1.49)	339	1.27	(0.91–1.76)
	≥13	54	0.81	(0.45–1.46)	54	0.73	(0.39–1.36)
Number of night work duties^c^	0	505	1		491	1	
	1–3	56	1.16	(0.64–2.12)	54	1.19	(0.64–2.21)
	4–6	83	1.00	(0.61–1.63)	81	1.09	(0.65–1.83)
	≥7	186	0.99	(0.69–1.41)	182	1.07	(0.72–1.58)
Number of weekend work duties^d^	0	484	1		469	1	
	1–4	236	1.09	(0.78–1.52)	229	1.19	(0.83–1.70)
	≥5	52	0.90	(0.50–1.63)	52	0.94	(0.50–1.75)
Number of ≤11-hour shift intervals	0	521	1		506	1	
	1–6	112	0.99	(0.65–1.54)	109	1.11	(0.71–1.75)
	7–12	98	0.89	(0.57–1.39)	95	1.02	(0.63–1.65)
	> 12	91	0.87	(0.54–1.38)	90	0.90	(0.55–1.48)
On-call work at home hours/week	0	461	1		451	1	
	1–20	182	1.20	(0.82–1.74)	176	1.31	(0.89–1.94)
	> 20	187	0.77	(0.54–1.09)	181	0.77	(0.53–1.11)

^a^multinomial logistic regression analysis

^b^adjusted for age, gender, marital status, number of children, overall stressfulness of the life situation, control over working times and hospital district

^c^at least 3 hours of work between 23 and 06

^d^at least 3 hours of work between Fri 18:00 and Mon 08:00

## Discussion

This study investigated the associations between hospital physicians’ realized working hours and on-call duties with sleep. Frequent night work and estimated long total weekly working hours were associated with short sleep duration. Both total long weekly working hours and several specific on-call working hour characteristics were associated with insufficient sleep. Even though nearly half of the physicians had often sleep difficulties, in a separate analysis, only frequent night work duties were associated with difficulties falling asleep.

### Working hour characteristics

The mean realized weekly working time of the hospital physicians was approximately 38 hours per week. The main challenge in comparison with earlier research is that previously the working hours have been self-reported, and it is known that self-reported hours may deviate from actual hours worked [3]. In our study, the total estimated weekly working hours were over 45 hours when also on-call work at home and possible second jobs were included in the estimations. It is likely that when answering a survey, a respondent thinks of a typical work week and does not calculate reductions for sickness or other absences from work. However, the self-reported weekly hours in this study were at the same level than previous national [34] or Nordic figs [3] and somewhat below European comparative estimated figures around 50 hours per week [1]. Compared to previous survey studies, the mean frequency of on-call shifts [6, 8, 9, 35, 36] and short shift intervals [37] were somewhat lower in this payroll data. On-call work at home was more common among physicians included in our study than in a previous study [38].

### Sleep length

In this study, approximately one fourth of the physicians reported short (≤6.5 h) habitual sleep duration. In previous studies, physicians’ average sleep durations have been at least 7 hours [12], but not when having had night-time duties [39]. Of the studied working time characteristics, only frequent night work was associated with short sleep. Previous studies [37, 40] on physicians’ night-time on-call duties have shown parallel results. For example, the connection between reduced sleep and consecutive [37, 39] or otherwise frequent [40] night duties as well as long working times [9]. Contradictory to a recent review [23], having on-call work at home for less than 20 hours per week was associated with lower odds for short sleep compared to those with no on-call work at home at all. As on-call work at home often takes place during night-time, we conducted an additional analysis with the amount of night work as a covariate, but this did not change the results. We speculate that that result may be related to the different seniority position of those having a limited amount of on-call work at home or could derive from less frequent calls to doctors than in an earlier study [10].

Overall, we observed only a few associations between working hour characteristics and sleep duration. It has been reported that although physicians´ longer work hours are associated with shorter sleep, very short sleep (< 5 h) is associated with different amounts of work [25]. However, previous studies have suggested that physicians prioritize other activities than sleep during their free time also when working fewer hours [4, 9].

### Sleep sufficiency

Working hour characteristics which were associated with perceived insufficient sleep included both realized and estimated long weekly working hours and a high number of on-call shifts, night work, and over 12 short shift intervals (< 11 h) during the preceding 3 months. The association was strongest between a high number of on-call duties and insufficient sleep. There are few previous studies that would have studied physicians’ working hour characteristics and sufficiency of sleep, but previous results confirm that insufficient/non-restorative sleep is commonly reported by physicians [8, 41] or physicians working both night-time and overtime duties [42]. However, straightforward conclusions regarding physicians’ preceding insufficient sleep and negative patient outcomes should not be drawn [41].

### Sleep difficulties

Nearly half of the physicians (48%) in this study reported having had sleep difficulties, which is a slightly higher [43, 44] or similar [42] proportion than in previous studies. Higher prevalence among female physicians has also been previously reported [43]. However, none of the studied working hour characteristics were associated with sleep difficulties. When the sleep difficulties were analysed separately, frequent night work increased odds for difficulties in initiating sleep, which is in line with previous studies on longer sleep latency in connection with night duties [42]. It can be speculated whether physicians pay enough attention to their sleep quality, as there are results showing that physicians may not use recommended good sleeping strategies, such as engaging in regular physical activity or seeking sleep specialist advice to improve sleep [42].

### Strengths and limitations

This study used physicians´ register-based working hour data to calculate accurate working hour characteristics for both regular and on-call hours. The use of register-based working hour data is the main strength of this study. Previous research has been conducted for the most part using self-reported working hour data which is prone to recall bias [26]. However, comparison to previous studies is limited due to methodological differences in working hour data collection (payroll data vs. survey data on working hours) and the sleep scale utilized. Another strength of this study was the relatively large sample covering both university and regional hospitals, and the comprehensive survey data that enabled multiple adjustments in the statistical models. Previous research has also often included junior doctors only [14, 25, 37, 45], whereas this data included physicians in different career stages.

There are limitations to be noted also. The working hour data was collected from all the physicians included in the payroll data in the studied four hospital districts, but the survey response was available for only less than 40% of these physicians. The physicians´ mean age and gender distribution were similar to the realized working hour data of all hospital physicians, but other sources of selection cannot be ruled out. On the other hand, 67% of the physicians with survey data had complete working hour data and were thus included to the final sample. The physicians without working hour data were for example chief physicians in administrative positions. The larger amount of on-call work at home in this study may indicate that these respondents had more senior positions than physicians in some of the previous studies [38]. There was a discrepancy between realized and self-estimated weekly working hours, which is likely being explained by the respondents´ tendency of not to subtract sickness or other absences from work from self-estimated average hours. Moreover, due to questionnaire wording, it is likely that also on-call work at home and work hours in possible second jobs were included.

Importantly, the employers’ register data did not allow us to differentiate between different medical specialties, and there may be differences in the amount and arrangement of on-call schedules. We used self-reported sleep data, which is subject to recall bias. The categorical variables for the outcomes limited the statistical analysis and the multinomial analysis we used may lack sensitivity to distinguish small differences. As there is variation in both legislation limiting, for example, the physicians’ maximum number of weekly working hours, and organization of health care services in different countries [46] these results are likely best generalizable to other European Union member states that comply with the European Working Time Directive [28].

As on-call duties are concentrated on certain medical specialities, such as anaesthesiologists [12, 17] and surgeons [6], it would be justified to study further on-call working hour characteristics and sleep among these specialists. Similarly to other occupations that require working in irregular shifts, studying sleep quality directly in connection with different shift or on-call characteristics would be merited. Extending precise data collection on active working hours and rest opportunities while on-call would be of great value as well.

## Conclusion

We used realized working hour data to strengthen the evidence on on-call work and sleep quality and our results advice to limit the frequency of night work, on-call shifts, short shift intervals and long weekly working hours to promote hospital physicians’ sufficient sleep.

## Supplementary Information

**Additional file 1: Supplementary Table 1.** The associations of physicians’ working time characteristics from 3 months with short sleep duration (≤6.5 h), insufficient sleep and having at least one sleep difficulty often with age, sex, and hospital district as covariates. **Supplementary Table 2.** The associations of physicians’ working time characteristics from 3 months with often having difficulties to fall asleep, waking up several times per night, difficulties in staying asleep and non-restorative sleep with age, sex, and hospital district as covariates.

## Data Availability

The research data are not publicly available due to privacy and ethical restrictions. Research data are not available on request from the authors or FIOH due to written agreements with the register data owners (the hospital districts) not to forward any data to third parties.
