# Supplementary material for: Hospital physicians´ working hour characteristics and sleep quality: a cross-sectional analysis of realized working hour and survey data
Source: BMC Health Serv Res. 2022 Jul 23;22:943. doi: 10.1186/s12913-022-08336-0 (PMC9308190; doi:10.1186/s12913-022-08336-0)
Supplement: Supplementary file 1 — Additional file 1: Supplementary Table 1. The associations of physicians’ working time characteristics from 3 months with short sleep duration (≤6.5 h), insufficient sleep and having at least one sleep difficulty often with age, sex, and hospital district as covariates. Supplementary Table 2. The associations of physicians’ working time characteristics from 3 months with often having difficulties to fall asleep, waking up several times per night, difficulties in staying asleep and non-restorative sleep with age, sex, and hospital district as covariates. [file 12913_2022_8336_MOESM1_ESM.docx]

Supplementary table 1. The associations of physicians’ working time characteristics from 3 months with short sleep duration (≤6.5h), insufficient sleep and having at least one sleep difficulty often with age, sex, and hospital district as covariates.

|  | | Short sleep duration | | | Insufficient sleep | | | At least one sleep difficulty | | |
| --- | --- | --- | --- | --- | --- | --- | --- | --- | --- | --- |
|  | | Adjusted model^1^ | | | Adjusted model^1^ | | | Adjusted model^1^ | | |
|  | | N | OR | 95%CI | N | OR | 95%CI | N | OR | 95%CI |
| Average working hours/week | <40 | 450 | 1 |  | 438 | 1 |  | 450 | 1 |  |
|  | 40–48 | 150 | 1.30 | (0.85–2.00) | 148 | 1.31 | (0.88–1.96) | 149 | 1.09 | (0.75–1.58) |
|  | >48 | 120 | 1.56 | (0.98–2.50) | 119 | 1.70 | (1.11–2.62) | 121 | 0.69 | (0.46–1.04) |
| Number of on-call shifts | 0 | 427 | 1 |  | 417 | 1 |  | 425 | 1 |  |
|  | 1–12 | 346 | 1.17 | (0.82–1.65) | 339 | 1.12 | (0.80–1.56) | 346 | 1.06 | (0.78–1.43) |
|  | ≥13 | 55 | 1.27 | (0.64–2.50) | 55 | 1.65 | (0.90–3.02) | 55 | 1.06 | (0.59–1.91) |
| Number of night work duties^3^ | 0 | 503 | 1 |  | 490 | 1 |  | 502 | 1 |  |
|  | 1–3 | 56 | 0.88 | (0.43–1.78) | 56 | 0.68 | (0.35–1.33) | 56 | 0.95 | (0.54–1.67) |
|  | 4–6 | 82 | 1.50 | (0.86–2.60) | 81 | 1.42 | (0.86–2.34) | 82 | 0.85 | (0.53–1.38) |
|  | ≥7 | 187 | 1.91 | (1.27–2.87) | 184 | 1.47 | (1.00–2.15) | 186 | 0.97 | (0.68–1.38) |
| Number of weekend work duties^4^ | 0 | 483 | 1 |  | 472 | 1 |  | 481 | 1 |  |
|  | 1–4 | 234 | 1.00 | (0.68–1.48) | 229 | 1.12 | (0.79–1.61) | 234 | 1.08 | (0.78–1.50) |
|  | ≥5 | 52 | 1.84 | (0.96–3.53) | 51 | 1.32 | (0.71–2.46) | 52 | 1.35 | (0.74–2.46) |
| Number of ≤11-hour shift intervals | 0 | 520 | 1 |  | 509 | 1 |  | 518 | 1 |  |
|  | 1–6 | 111 | 0.77 | (0.47–1.29) | 108 | 1.09 | (0.69–1.73) | 111 | 1.10 | (0.72–1.67) |
|  | 7–12 | 97 | 1.18 | (0.70–1.97) | 94 | 1.47 | (0.92–2.35) | 98 | 0.82 | (0.53–1.28) |
|  | ≥13 | 92 | 1.10 | (0.64–1.88) | 92 | 1.51 | (0.93–2.45) | 92 | 0.86 | (0.54–1.37) |
| On-call work at home hours/week | 0 | 459 | 1 |  | 452 | 1 |  | 458 | 1 |  |
|  | 1–20 | 181 | 0.72 | (0.47–1.09) | 174 | 0.75 | (0.50–1.11) | 181 | 1.05 | (0.47–1.50) |
|  | >20 | 188 | 1.06 | (0.72–1.55) | 185 | 1.01 | (0.69–1.46) | 187 | 0.84 | (0.59–1.18) |

^1^ multinomial regression analysis adjusted for age, gender, and hospital district

^2^ at least 3 hours of work between 23–06

^3^ at least 3 hours of work between Fri 18:00 and Mon 08:00

Supplementary table 2. The associations of physicians’ working time characteristics from 3 months with often having difficulties to fall asleep, waking up several times per night, difficulties in staying asleep and non-restorative sleep with age, sex, and hospital district as covariates.

|  | | Difficulties to fall asleep | | | Waking up several times per night | | | Having difficulties in staying asleep | | | Non-restorative sleep | | |
| --- | --- | --- | --- | --- | --- | --- | --- | --- | --- | --- | --- | --- | --- |
|  | | Adjusted model^1^ | | | Adjusted model^1^ | | | Adjusted model^1^ | | | Adjusted model^1^ | | |
|  | | N | OR | N | N | OR | 95%CI | N | OR | 95%CI | N | OR | 95%CI |
| Average working hours/week | <40 | 450 | 1 |  | 450 | 1 |  | 448 | 1 |  | 451 | 1 |  |
|  | 40–48 | 151 | 1.14 | (0.58–2.23) | 149 | 1.01 | (0.65–1.55) | 150 | 1.15 | (0.69–1.90) | 151 | 1.09 | (0.74–1.62) |
|  | >48 | 120 | 2.24 | (0.86–5.87) | 120 | 0.66 | (0.42–1.04) | 121 | 1.04 | (0.58–1.83) | 120 | 0.77 | (0.51–1.18) |
| Number of on-call shifts | 0 | 427 | 1 |  | 426 | 1 |  | 424 | 1 |  | 428 | 1 |  |
|  | 1–12 | 347 | 1.38 | (0.80–2.40) | 346 | 1.03 | (0.74–1.45) | 346 | 1.18 | (0.79–1.79) | 348 | 1.24 | (0.90–1.71) |
|  | ≥13 | 55 | 5.49 | (0.73–41.52) | 55 | 1.33 | (0.65–2.73) | 55 | 0.98 | (0.43–2.23) | 54 | 0.86 | (0.47–1.58) |
| Number of night work duties^2^ | 0 | 504 | 1 |  | 503 | 1 |  | 500 | 1 |  | 505 | 1 |  |
|  | 1–3 | 56 | 1.76 | (0.53–5.90) | 56 | 0.85 | (0.46–1.58) | 56 | 1.30 | (0.59–2.87) | 56 | 1.28 | (0.70–2.34) |
|  | 4–6 | 83 | 0.85 | (0.39–1.83) | 82 | 0.88 | (0.51–1.51) | 83 | 0.74 | (0.41–1.34) | 83 | 1.12 | (0.68–1.85) |
|  | ≥7 | 186 | 2.22 | (1.00–4.91) | 186 | 0.94 | (0.62–1.42) | 186 | 1.24 | (0.74–2.07) | 186 | 1.05 | (0.72–1.54) |
| Number of weekend work duties^3^ | 0 | 483 | 1 |  | 482 | 1 |  | 480 | 1 |  | 484 | 1 |  |
|  | 1–4 | 236 | 1.52 | (0.79–2.85) | 235 | 0.95 | (0.66–1.37) | 234 | 0.93 | (0.60–1.45) | 236 | 1.20 | (0.85–1.69) |
|  | ≥5 | 52 | 1.59 | (0.47–5.43) | 52 | 2.11 | (0.91–4.87) | 52 | 1.22 | (0.49–3.04) | 52 | 0.95 | (0.51–1.76) |
| Number of ≤11-hour shift intervals | 0 | 520 | 1 |  | 519 | 1 |  | 517 | 1 |  | 521 | 1 |  |
|  | 1–6 | 112 | 0.89 | (0.44–1.79) | 112 | 1.24 | (0.76–2.04) | 110 | 0.89 | (0.51–1.53) | 112 | 1.06 | (0.68–1.65) |
|  | 7–12 | 98 | 1.86 | (0.71–4.89) | 97 | 0.74 | (0.45–1.20) | 98 | 0.81 | (0.45–1.46) | 98 | 0.99 | (0.62–1.57) |
|  | ≥13 | 91 | 2.06 | (0.71–5.99) | 92 | 0.86 | (0.51–1.46) | 92 | 0.94 | (0.49–1.82) | 91 | 0.89 | (0.55–1.44) |
| On-call work at home hours/week | 0 | 461 | 1 |  | 460 | 1 |  | 457 | 1 |  | 461 | 1 |  |
|  | 1–20 | 180 | 1.46 | (0.70–3.01) | 180 | 0.71 | (0.48–1.04) | 182 | 0.95 | (0.60–1.51) | 182 | 1.21 | (0.83–1.76) |
|  | >20 | 188 | 0.75 | (0.42–1.35) | 187 | 0.84 | (0.57–1.24) | 186 | 0.78 | (0.50–1.21) | 187 | 0.73 | (0.51–1.04) |

^1^ multinomial regression analysis adjusted for age, gender, and hospital district

^2^ at least 3 hours of work between 23–06

^3^ at least 3 hours of work between Fri 18:00 and Mon 08:00
